# Effects of intermittent hemodialysis on plasmatic levels of endocan

**DOI:** 10.1186/s13054-021-03829-6

**Published:** 2021-11-29

**Authors:** Maxence Hureau, Julien Poissy, Daniel Mathieu, Sylvain Dubucquoi, Alexandre Gaudet

**Affiliations:** 1 Univ. Lille, U1019 – UMR 9017 – CIIL – Center for Infection and Immunity of Lille, 59000 Lille, France; 2 CNRS, UMR 9017, 59000 Lille, France; 3 INSERM, U1019, 59000 Lille, France; 4 CHU Lille, Pôle de Médecine Intensive – Réanimation, Hôpital Roger Salengro, 59000 Lille, France; 5 Univ. Lille, Inserm U1285, CHU Lille, CNRS, UMR 8576, UGSF, Unité de Glycobiologie Structurale et Fonctionnelle, 59000 Lille, France; 6 Univ. Lille, Inserm, CHU Lille, U1286 - INFINITE - Institute for Translational Research in Inflammation, 59000 Lille, France

To the editor,

Endocan is a prognostic biomarker of pulmonary and systemic inflammatory states [1], such as acute respiratory distress syndrome or sepsis, with substantial proportion of subjects requiring RRT [2]. In previous correspondences, Honoré et al. raised the question of the actual impact of hemodialysis on plasmatic levels of endocan in critically ill subjects [3, 4].

To address this question, we retrospectively collected serial measurements of endocan performed for routine care in EDTA plasma from 11 patients undergoing intermittent hemodialysis in a 50-bed ICU in Lille, France. Hemodialysis was performed using either ELISIO (Nipro, France), a polyethersulfone membrane with very low adsorptive capacities or EVODIAL (Baxter, USA), a heparin-grafted AN 69 ST membrane with higher adsorptive properties. Patients’ characteristics on ICU admission and on day of hemodialysis were collected retrospectively.

We categorized measurements of plasma endocan according to timing of blood collection relatively to hemodialysis, as following: T0, within 1 h before start of hemodialysis; T1, 30 min to 60 min after start of hemodialysis; T2, 90 min to 120 min after start of hemodialysis, T3, 180 min to 240 min after start of hemodialysis; T4: 90 min to 120 min after end of hemodialysis.

We are reporting characteristics of patients and of hemodialysis in Table 1 and kinetics of plasma endocan during hemodialysis in Fig. 1. We find a significant variation of endocan during the time course of hemodialysis, with median [IQR] values measured at 10.9 [4.3; 14.9] ng/ml at T0, 15.4 [5; 19.4] ng/ml at T1, 14.9 [5.4; 18.8] ng/ml at T2, 12.4 [6.1; 20] ng/ml at T3, 10.1 [4.2; 16.1] ng/ml at T4 (*p* = 0.025). In addition, relatively to values observed at T0, we found significant increases of median [IQR] plasma endocan variations at T1 (+ 22% [+ 12%; + 72%], *p* = 0.006), T2 (+ 34% [-4%; + 68%], *p* = 0.019), and T3 (+ 27% [+ 5%; + 56%], *p* = 0.014), but not at T4 (+ 34% [-8%; + 57%], *p* = 0.053). Noteworthy, our analyses suggest that the variations of endocan depend on the type of membrane, with lower increases in the EVODIAL group (*p* = 0.009 by linear mixed model). This lower increase in endocan blood levels seems consistent with greater adsorption properties of EVODIAL membranes. However, because of the limited number of patients in the EVODIAL group, this result should be interpreted with caution.

**Table 1:** Characteristics of patients

Sex, male	6 (55%)
Age, years	66 (59–69)
Body weight on ICU admission, kg	80 (72–96)
SAPS II on ICU admission	48 (41–72)
Disorder category	
Medical	8 (73%)
Surgical	3 (27%)
Preexisting conditions	
Chronic kidney failure	5 (45%)
Chronic heart failure	3 (27%)
Cirrhosis	1 (9%)
Diabetes mellitus	4 (36%)
Diagnosis on ICU admission	
Soft tissues infection	3 (27%)
Pneumonia	3 (27)
Carbon monoxide poisoning	1 (9%)
Decompensated cirrhosis	1 (9%)
Cardiogenic shock	1 (9%)
Hemorrhagic shock	1 (9%)
Gas embolism	1 (9%)
Inflammation biomarkers on day of hemodialysis	
CRP, mg/l	66 (41–154)
Procalcitonin, ng/ml	2 (1.4–2.8)
Characteristics of hemodialysis	
Residual volume of diuresis (ml/24 h)	100 (25–450)
Systemic anticoagulation	8 (72%)
Daily UFH dose, IU/24 h	10000 (0; 12000)
Heparin-coated membrane (EVODIAL)	3 (27%)
Duration of hemodialysis, hrs	5 (4.5–6)
Blood flow rate, ml/min	250 (250–250)
Dialysate flow rate, ml/min	500 (500–500)
Ultrafiltration volume, ml	2000 (860–2500)
Ultrafiltration rate, ml/h	333 (240–500)

Anticoagulation of the extracorporeal circuit with unfractioned heparin 5000 IU was performed for all patients. Data are presented as number (%) or median (IQR)

SAPS, Simplified Acute Physiology Score; ICU, Intensive Care Unit; IU, International Unit; UFH, unfractioned heparin

**Fig. 1:**
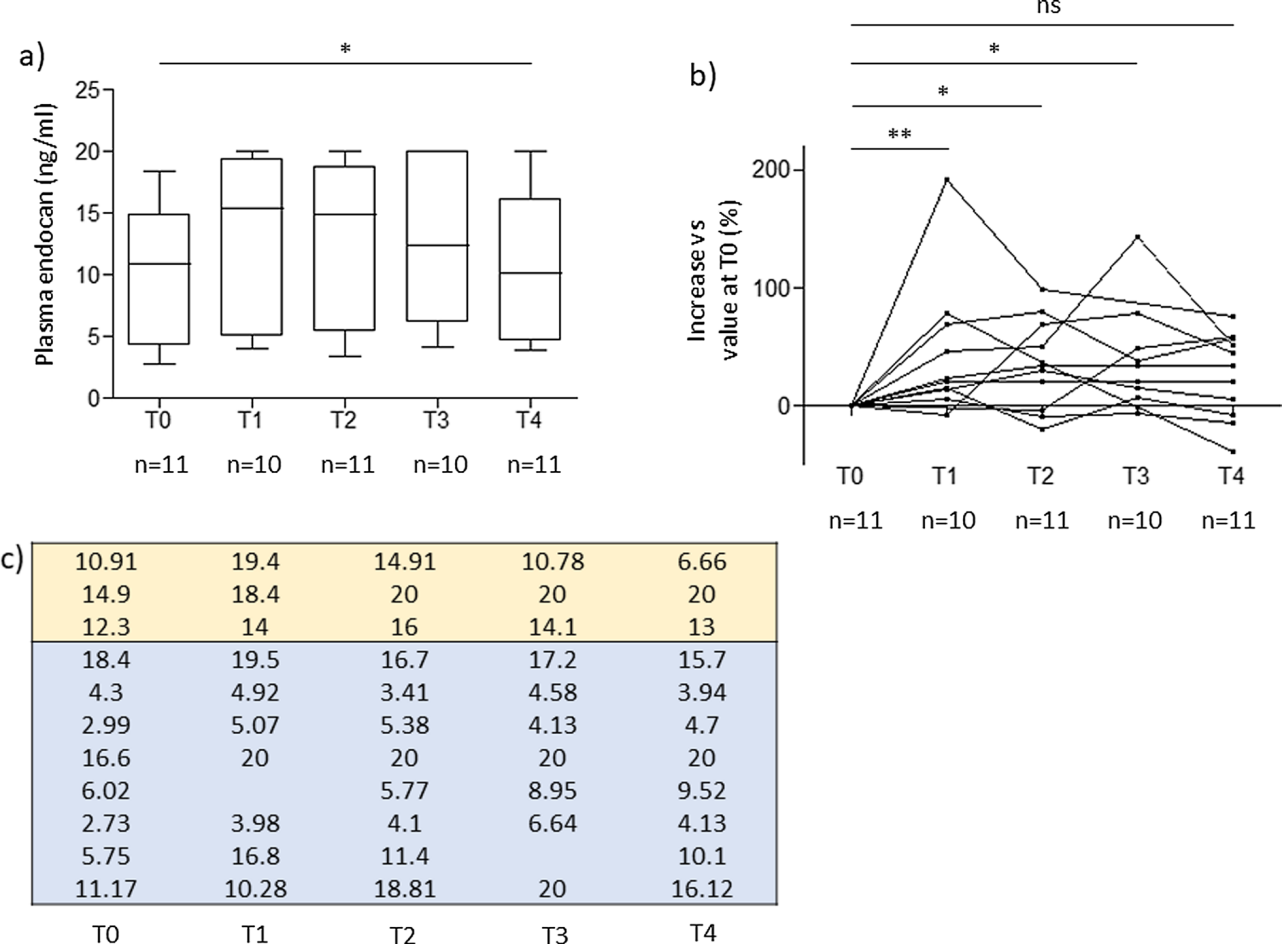
**a** Box plots of plasma endocan values during hemodialysis. Box plots show median, 1st and 3rd quartiles, and whiskers at 1.5-time interquartile range. Comparisons between paired values were performed using Friedman test. **b** Variations of plasma endocan relatively to values measured at T0. Comparisons between values at each time point with T0 value were performed using Wilcoxon-signed ranks test. **c** Values of endocan expressed in ng/ml at each time point. Patients are shown in yellow rows in case of hemodialysis with EVODIAL membranes and blue rows in case of ELISIO membranes. We used a linear mixed model with type of membrane set as fix effect and timing of blood collection set as random effect to assess the effect of the type of membrane on variations of endocan during hemodialysis. Plasma endocan was measured at the Immunology Institute of Lille Teaching Hospital, France, using the ENDOMARK H1 ELISA kit (Biothelis, France). T0: sampling within 1 h before start of hemodialysis. T1: sampling 30–60 min after start of hemodialysis. T2: sampling 90–120 min after start of hemodialysis. T3: sampling 180–240 min after start of hemodialysis. T4: sampling 90–120 min after end of hemodialysis. All statistical tests were two-tailed, and *p* values < 0.05 were considered statistically significant. All data analyses were performed using R, version 3.6 (R Foundation for Statistical Computing, Austria). *p < 0.05. **p < 0.01. ns: non-significant

Our results suggest an increase in levels of plasma endocan at the initial phase of hemodialysis. This may be explained by the response to vascular stress, resulting in an increase in secretion of endocan [1], or by hemoconcentration, which may occur during hemodialysis. Interestingly, the quick raise in endocan blood levels is consistent with previous data from a human LPS-induced endothelial stress model [5]. Additionally, our data tend to show a progressive decrease of plasma endocan during the time course of hemodialysis, possibly related to alleviation of endothelial stress or actual elimination of endocan. Adsorption on the membrane may explain this progressive decrease in blood levels of endocan, as suggested by the likely influence of adsorptive properties of the membranes on the variations of endocan. Conversely, the possibility that this decrease could be explained by diffusion mechanisms seems unlikely, given the 50 kDa molecular weight of endocan, exceeding the diffusion properties expected with our hemodialysis settings [6]. Similar mechanisms should be observed during continuous renal replacement therapy (CRRT), potentially resulting in greater removal of plasma endocan, because of longer RRT durations in CRRT.

Hence, these results suggest major interference of hemodialysis with blood concentrations of endocan, especially when highly adsorptive membranes are used, making it unreliable as a prognostic biomarker of pulmonary and systemic inflammation in critically ill patients undergoing hemodialysis.

## Data Availability

The datasets used and/or analyzed during the current study are available from the corresponding author on reasonable request.
